# The treatment-related mortality score is associated with non-fatal adverse events following intensive AML induction chemotherapy

**DOI:** 10.1038/bcj.2014.97

**Published:** 2015-01-30

**Authors:** S A Buckley, M Othus, E H Estey, R B Walter

**Affiliations:** 1 Hematology/Oncology Fellowship Program, University of Washington, Seattle, WA, USA; 2 Public Health Sciences Division, Fred Hutchinson Cancer Research Center, Seattle, WA, USA; 3 Department of Medicine, Division of Hematology, University of Washington, Seattle, WA, USA; 4 Clinical Research Division, Fred Hutchinson Cancer Research Center, Seattle, WA, USA; 5 Department of Epidemiology, University of Washington, Seattle, WA, USA

The risk of death during intensive remission induction therapy (‘treatment-related mortality' TRM) for adults with acute myeloid leukemia (AML) has substantially decreased over the last two decades,^1^ likely largely because of improved supportive care measures. Still, AML patients remain prone to morbidity during the prolonged duration of disease- and treatment-related cytopenia, especially with regard to the development of infections.^2^ Identifying the subset of patients at particularly high risk of experiencing non-fatal adverse events following induction chemotherapy could not only help in advising patients more accurately about the likelihood of encountering such events but also in allocating appropriate health-care resources toward these higher-risk individuals.

Several groups, including ours, have proposed algorithms to identify patients suited for intensive AML therapy.^3^ Our efforts led to the development of the TRM score—a multivariable score that includes weighted information on age, performance status, disease type (primary vs secondary AML), creatinine, white blood cell (WBC) count, peripheral blood blast percentage, platelet count and albumin in its ‘simplified' version. Using area under receiver operator characteristic curves (AUCs), we demonstrated that this score allows the prediction of death within 28 days of treatment initiation (our empiric definition of TRM) with good accuracy (AUC=0.82, with AUC=1 denoting perfect predictive ability and AUC=0.5 denoting no predictive ability).^4^ Although the TRM score was established primarily as a tool for the assignment of appropriate treatment intensity based on the likelihood of experiencing fatal treatment-related complications, we reasoned that this score might also be useful in identifying patients at high risk of non-fatal adverse events. Here, we tested this assumption.

To this end, we retrospectively analyzed all 179 adults aged ⩾18 years with newly diagnosed AML treated with curative-intent chemotherapy with ‘7+3' or a ‘7+3'-like regimen between August 2002 and August 2012 at our institution for whom we had complete inforrmation to calculate the TRM score. The ‘simplified' score was calculated using the formula: 100/(1+*e*^(-*x*)^) with *x*=−4.08+0.89 × performance status+0.03 × age–0.008 × platelets–0.48 × albumin+0.47 × have secondary AML+0.007 × WBC–0.007 × peripheral blood blast percentage+0.34 × creatinine.^4^ Medical records were further reviewed to obtain information on demographics and disease characteristics as well as on treatment regimens and outcomes, including adverse events such as death, fever (as defined by the Infectious Diseases Society of America^5^), infection (defined on microbiological and clinical grounds^6^) and requirement for intensive care unit (ICU)-level care. All adverse outcomes were recorded until the earlier of day 35 or administration of additional chemotherapy; however, as we had previously identified the first 28 days as the period of interest for TRM events,^4^ data were censored at day 28 for our analyses. The survival probabilities in the absence of infection or ICU transfer were estimated using the Kaplan–Meier method; the 10 patients in the ICU at the start of chemotherapy were excluded from analysis of ICU transfer as an adverse event. Patients were categorized at baseline by quartiles of TRM score, and outcomes between quartiles were assessed using the log-rank test for trend; scores above and below median were compared using Cox regression. Multivariable models were adjusted for gender, cytogenetic risk as defined by the Modified Medical Research Council criteria,^7^ baseline absolute neutrophil count^8^ and year of treatment.

Baseline characteristics of our study cohort are summarized in Table 1. The median TRM score was 4.67, with 2.3 and 10.5 being the boundaries for quartiles 1/2 and 3/4, respectively. Documented infections occurred in 72 patients (40%). ICU transfer was necessary in 14 (8%) patients, primarily for the treatment of sepsis (*n*=8) or respiratory distress (*n*=3), with other reasons being cardiogenic shock, encephalitis and epistaxis. Of these 14 patients, 4 (2%) died within 28 days of initiation of induction chemotherapy; no patient experienced TRM without being transferred to the ICU first.

When patients were stratified by quartiles of TRM scores, higher scores were associated with development of infections (*P*_*trend*_=0.006; Figure 1a) and requirement for ICU transfer (*P*_*trend*_=0.003, Figure 1b), but not development of fever (*P*_*trend*_=0.247). On the basis of these findings, we subsequently dichotomized patients by the median of TRM scores, and found that patients with TRM scores above the median had an increased risk of infection (*P*=0.02; Figure 1c), ICU transfer (*P*=0.0004; Figure 1d) and death (*P*=0.046), but not of developing fever (*P*=0.63). Noticeably, among the patients with lower TRM scores (that is, <median), only one required ICU transfer (for respiratory distress due to diffuse alveolar hemorrhage), and no deaths occurred. After multivariable adjustment, the risk of documented infection was 1.65 (95% confidence interval: 1.02–2.67)-fold higher for patients with higher TRM scores (that is, >median). In developing the TRM score, it was noted that the death rate peaked 3–4 weeks after the start of treatment and fell sharply after day 28, suggesting that most TRM occurred within this timeframe. A similar trend was observed in our cohort with regard to non-fatal events, which mostly occurred within the first 3 weeks of treatment. For example, of the 72 patients who developed infection, 97% (*n*=70) did so within 3 weeks while only two and one additional patients developed infection between weeks 3 and 4 and between weeks 4 and 5, respectively. Likewise, of the 14 ICU transfers, all but one (93%) occurred within 3 weeks of the start of treatment, with only one patient requiring ICU transfer between weeks 3 and 4, and no transfers occurred between weeks 4 and 5.

As TRM rates have significantly decreased over the years,^1^ we investigated whether the TRM score is still of value in identifying current subsets of patients at high risk of non-fatal adverse events. Indeed, restricting the dataset to patients undergoing therapy between 2007 and 2012, a high TRM score (>median) remained associated with the requirement for ICU-level care (*P*=0.001), although there was no longer a significantly higher risk of developing a documented infection (*P*=0.319). The reason for the latter observation remains currently unclear and it remains speculative whether changes in antimicrobial prophylaxis could account for (or contribute to) this finding.

In conclusion, our data indicate that the TRM score is associated not only with fatal but also non-fatal early adverse events following intensive induction chemotherapy for patients with newly diagnosed AML. Previous studies in patients with AML^8^ and other malignancies^9^ have identified baseline neutropenia, lymphopenia and monocytopenia as risk factors for the development of febrile neutropenia and documented infection. The TRM score, which incorporates information on pre-treatment peripheral blood WBC counts, is associated with both infection and ICU transfer in our cohort. Though our study is limited by its retrospective nature and the relatively small number of patients studied (explained by the fact that many of our patients with newly diagnosed AML receive induction therapy with high-dose cytarabine-based regimens rather than ‘7+3' or a ‘7+3'-like regimen), it does suggest that the TRM score can separate low- from high-risk patients in terms of susceptibility to infection and likelihood of requiring ICU transfer. For example, only 12 out of 45 patients in the lowest TRM score quartile (TRM score <2.3) developed an infection (26.7%), and none required ICU transfer. On the other hand, 25 out of 45 patients in the highest TRM score quartile (TRM >10.5) developed an infection (55.6%), and 6 (13.3%) required ICU transfer (5 for sepsis). Patients in the second and third quartiles of TRM scores in our cohort had an intermediate risk of developing infection. Their risk of infection requiring ICU care diverged based on TRM scores, with those in the second quartile (TRM 2.31–4.54) having very low risk for ICU transfer (0 ICU transfers for sepsis) and those in the third quartile (TRM 4.67–10.25) having a higher rate of severe infection (4 ICU transfers for sepsis). Although the optimal cut-point may need to be determined in future studies in independent patient cohorts and may need to be re-calibrated periodically with ongoing improvements in supportive care, our data overall suggest that the TRM score could be useful as a tool to help clinicians improve their assessment of the risks of intensive induction chemotherapy, communicate these risks accurately to patients and allocate the health-care resources accordingly.

## Figures and Tables

**Figure 1:**
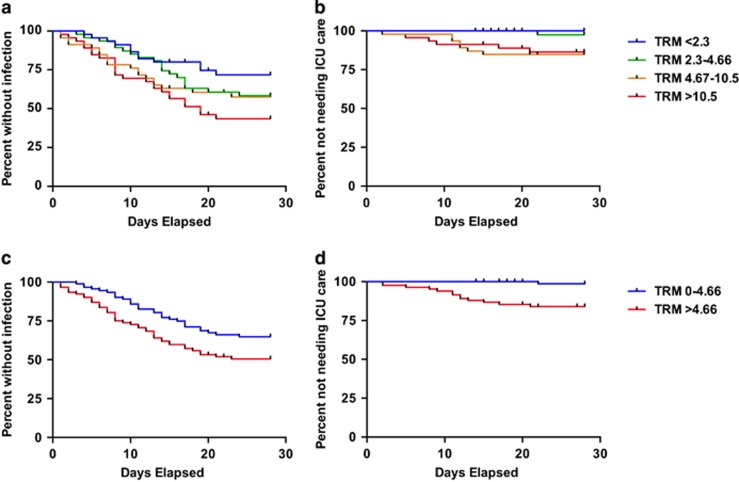
Higher TRM scores are associated with early non-fatal adverse events after AML induction therapy. Kaplan–Meier estimates of freedom from infection (**a**,**c**) and requirement for ICU care (**b**,**d**) in our cohort, stratified by quartiles or median of TRM score, from the first day of induction chemotherapy until day 28; patients who received salvage chemotherapy were censored on the first day of initiation of such therapy. In our cohort, high TRM scores were significantly associated with infection (*P*_*trend*_=0.006) and ICU transfer (*P*_*trend*_=0.003), particularly when the cohort was stratified by median TRM score of 4.67 (*P*=0.02, *P*=0.0004, respectively).

**Table 1:** Basic characteristics of study cohort

P*arameter*	*All patients* n=*179*	*First quartile* n=*43*	*Second quartile* n=*46*	*Third quartile* n=*45*	*Fourth quartile* n=*45*
Age (years), median (range)	53 (18–77)	45 (18–65)	51.5 (25–69)	57 (19–76)	60 (22–77)
Male gender, *n* (%)	98 (55)	19 (44.2)	22 (47.8)	30 (66.7)	27 (60.0)
					
*Category of disease,* n *(%)*					
Primary AML	119 (66.5)	33 (76.7)	35 (76.1)	21 (46.7)	30 (66.7)
Secondary AML	60 (33.5)	10 (23.3)	11 (23.9)	24 (53.3)	15 (33.3)
					
*Disease risk*^a^, n *(%)*					
Favorable	27 (15.1)	6 (14.0)	9 (19.6)	5 (11.1)	7 (15.6)
Intermediate	108 (60.3)	30 (70.0)	26 (56.5)	30 (66.7)	22 (48.9)
Adverse	41 (22.9)	7 (16.3)	10 (21.7)	10 (22.2)	14 (31.1)
Missing	3 (1.7)	0 (0)	1 (2.2)	0 (0)	2 (4.4)
TRM score, median (range)	4.67 (0.15–77.50)	1.46 (0.15–2.28)	3.18 (2.31–4.54)	6.13 (4.67–10.25)	23.36 (10.81–77.50)
					
*Performance status,* n *(%)*					
0–1	132 (73.7)	42 (97.7)	46 (100)	37 (82.2)	7 (15.6)
2	15 (8.4)	0 (0)	0 (0)	6 (13.3)	9 (0.2)
3	18 (10.1)	1 (2.3)	0 (0)	1 (2.2)	16 (35.6)
4	14 (7.8)	0 (0)	0 (0)	1 (2.2)	13 (28.9)
Total WBC ( × 10^3^/μl)	13.1 (0.1–372.0)	16.1 (0.7–76.0)	5.4 (0.5–92.2)	3.2 (0.2–131.4)	31.9 (0.1–372.0)
% Peripheral blood blasts	25.9 (0–96.0)	49.0 (0–92.0)	15.1 (0–94.0)	15.8 (0–96.0)	42.0 (0–93.0)
Platelets ( × 10^3^/μl)	50 (5–547)	106 (17–547)	48.5 (12–215)	46 (5–138)	40 (8–93)
Creatinine (mg/dl)	0.9 (0.42–4.26)	0.8 (0.5–1.5)	0.83 (0.6–1.6)	0.9 (0.42–2.90)	1.2 (0.5–4.26)
Albumin (g/dL)	3.2 (1.4–4.6)	3.5 (2.2–4.5)	3.5 (1.9–4.6)	3.1 (2.1–4.1)	2.7 (1.4–4.1)

Abbreviations: AML, acute myeloid leukemia; TRM, treatment-related mortality.

Treatment regimens included 7+3 (*N*=150), 7+3+gemtuzumab ozogamicin (*N*=14), 7+3+cladribine (*N*=2), 7+3+sorafenib (*N*=3), 7+3+demethylating agent (*N*=3), 7+3+high-dose pravastatin (*N*=2) and 7+3+all-*trans* retinoic acid (*N*=5).

a Risk based on modified Medical Research Council (MRC) criteria.
